# Trends of the Major Porin Gene (*ompF*) Evolution: Insight from the Genus *Yersinia*

**DOI:** 10.1371/journal.pone.0020546

**Published:** 2011-05-31

**Authors:** Anna M. Stenkova, Marina P. Isaeva, Felix N. Shubin, Valeri A. Rasskazov, Alexander V. Rakin

**Affiliations:** 1 Pacific Institute of Bioorganic Chemistry, Far Eastern Branch of Russian Academy of Sciences, Vladivostok, Russian Federation; 2 Scientific Research Institute of Epidemiology and Microbiology, Siberian Branch of Russian Academy of Medical Sciences, Vladivostok, Russian Federation; 3 Max von Pettenkofer Institute for Hygiene and Clinical Microbiology of Ludwig Maximilians-University, Munich, Germany; University of Birmingham, United Kingdom

## Abstract

OmpF is one of the major general porins of *Enterobacteriaceae* that belongs to the first line of bacterial defense and interactions with the biotic as well as abiotic environments. Porins are surface exposed and their structures strongly reflect the history of multiple interactions with the environmental challenges. Unfortunately, little is known on diversity of porin genes of *Enterobacteriaceae* and the genus *Yersinia* especially. We analyzed the sequences of the *ompF* gene from 73 *Yersinia* strains covering 14 known species. The phylogenetic analysis placed most of the *Yersinia* strains in the same line assigned by *16S rDNA-gyrB* tree. Very high congruence in the tree topologies was observed for *Y. enterocolitica, Y. kristensenii, Y. ruckeri,* indicating that intragenic recombination in these species had no effect on the *ompF* gene. A significant level of intra- and interspecies recombination was found for *Y. aleksiciae, Y. intermedia* and *Y. mollaretii*. Our analysis shows that the *ompF* gene of *Yersinia* has evolved with nonrandom mutational rate under purifying selection. However, several surface loops in the OmpF porin contain positively selected sites, which very likely reflect adaptive diversification *Yersinia* to their ecological niches. To our knowledge, this is a first investigation of diversity of the porin gene covering the whole genus of the family *Enterobacteriaceae*. This study demonstrates that recombination and positive selection both contribute to evolution of *ompF*, but the relative contribution of these evolutionary forces are different among *Yersinia* species.

## Introduction

The genus *Yersinia*, a member of the *Enterobacteriaceae* family, is currently composed of 14 known species: *Y. pestis*, *Y. pseudotuberculosis*, *Y. enterocolitica Y. aldovae*, *Y. aleksiciae*, *Y. bercovieri*, *Y. frederiksenii*, *Y. intermedia*, *Y. kristensenii*, *Y. massiliensis*, *Y. mollaretii*, *Y. rohdei*, *Y. ruckeri*, and *Y. similis* [1]–[3]. Three of them are well documented human pathogens. *Y. pestis* is the etiologic agent of plague while *Y. pseudotuberculosis* and *Y. enterocolitica* are known to cause a variety of gastrointestinal symptoms [4]. The characterization of the remaining 11 species is more limited. However, these species accepted as human nonpathogenic possess novel virulence mechanisms, and some of them have been associated with human cases [5], [6]. *Yersinia* are disseminated all over the world in terrestrial and aquatic environments, and associated with many different hosts (plants, animals, insects, fish and so on). Despite recent advances in our understanding of the pathobiology of *Yersinia*, the molecular-genetic mechanisms by which *Yersinia* colonizes and adapts to various host or environmental conditions are still poorly understood. In this context, membrane surface molecules are considered the major targets of the membrane-environment interaction.

General bacterial porins (GBPs) are one of the most abundant proteins (up to 10^5^ copies per cell) in the outer membrane of the gram-negative bacteria [7], [8]. Structurally, a typical GBP subunit consists of 16 antiparallel β-strands forming a β-barrel, with short turns facing the periplasmic space and long loops facing the external surface of bacterial membrane [9]–[11]. Three porin subunits are assembled into stable homotrimers. The best-studied GBPs, which include OmpF, OmpC and PhoE of *E. coli*, differ in their solute selectivity, porin activity and gene expression in response to many environmental factors, such as osmotic pressure, temperature and pH [12]–[14]. Porins are one of the first molecules responding to environmental changes and at least for some bacteria have been found to reflect their ecological niche by the sequence type [15], [16]. As the major components of the outer membrane, some pore-forming proteins play a role in bacterial pathogenesis, such as adherence, invasion, and serum resistance [17]–[20].

Little is known about evolution and diversity of GBPs of the *Enterobacteriaceae* at all and the *Yersinia* especially. Scattered reports showed that *Yersinia's* major porin is the β-structured protein resistant to high temperature, proteases, and detergents [21]–[23]. Primary structure and topology of the OmpF porin of pathogenic *Yersinia* was determined and demonstrated 55% homology with *E. coli* and 70% homology with *Serratia marcescens* OmpFs, respectively [24], [25]. Here we conducted an in-depth study of the *ompF* gene diversity in all currently known *Yersinia* paying special attention to evolution inference and phylogenetic relationships of these bacteria.

## Results and Discussion

### 16S rDNA and gyrB sequence variations and genetic relationships among Yersinia species

To justify evolutionary relationships and taxonomic position, 16S rDNA and *gyrB* genes sequences were analysed in all *Yersinia* strains used in this study (Table 1). The 16S rDNA gene sequencing has definitely allowed *Yersinia* identification [26] and recognizing novel species and subspecies within the genus [3], [27], [28]. However, the 16S rDNA sequence analysis cannot resolve the phylogenetic relationships between closely related *Yersinia* species [2], [29]. Recently, *gyrB* has been successfully applied to characterization of *Y. frederiksenii* genomospecies [30] and was included as one of the MLST gene targets for studying genetic relationships among *Yersinia* species [29].

**Table 1:** *Yersinia* strains and the distribution of their 16S rDNA, *gyrB* and *ompF* gene alleles.

Species	Strain	Serotype	Source	Country	Allele type (NT/AT)^A^		
					16S RNA	*gyrB*	*ompF*
*Y. aldovae*	Y112				1	16/11	10/14
	ATCC 35236		Water	Czechoslovakia	1	46/20	58/14
*Y. aleksiciae*	Y159			Germany	2	15/1	11/15
*Y. bercovieri*	ATCC 43970		Human feces	France	17	1/2	13/16
	H632-36/85				14	1/2	12/17
*Y. enterocolitica* subsp. *palearctica*	Y11	O:3			3	2/3	1/1
	1234	O:3		Russia	3	3/4	15/18
	2974/81	O:9			3	2/3	17/1
	6579	O:3		Russia	3	2/3	1/1
	1245		Human feces	Russia	3	3/4	19/2
	2720/87	O:9			3	17/3	16/1
	1215		Human feces	Russia	3	3/4	14/2
subsp. *enterocolitica*	WA220	O:8			4	4/4	2/3
	ATCC 8081	O:8	Human	USA	4	4/4	18/19
*Y. frederiksenii*	H56-36/81	O:60		Germany	5	18/5	20/20
	4648		Human feces	Russia	5	5/6	22/21
	4849			Russia	5	20/13	24/22
	ATCC 33641		Sewage	Denmark	18	19/12	21/23
	176–36				19	5/6	23/24
*Y. massiliensis* B	2043			Russia	20	21/14	25/25
*Y. intermedia*	5631		Lemming	Russia	1	6/7	37/26
	5934		Citellus	Russia	1	14/7	30/27
	6325		Lemming	Russia	1	27/7	6/7
	ATCC 29909		Human urine		6	7/7	28/28
	5373		Water	Russia	6	6/7	¾
	6390		Lemming	Russia	6	9/7	5/6
	5593		Lemming	Russia	6	24/16	5/6
	5986		Field mouse	Russia	6	7/7	34/29
	H357/85	O:3			6	10/7	35/30
	Nr27/84	52,53:2q	Water	Germany	6	9/7	5/6
	H9-36/83	O:17		Germany	6	7/7	26/31
	Nr13/84	37:q	Human	Germany	7	8/7	27/32
	1948		Water	Russia	7	25/7	4/5
	5828		Field mouse	Russia	7	8/7	36/33
	6043			Russia	7	26/7	38/34
	5375		Water	Russia	7	8/7	4/5
	5638		Lemming	Russia	7	6/7	¾
	6270		Lemming	Russia	13	14/7	29/35
	6044		Field mouse	Russia	13	23/15	33/36
	Nr9/83	17:q	Human	Germany	21	10/7	31/37
	601			Russia	22	22/7	32/38
	6276		Lemming	Russia	23	28/17	6/7
*Y. kristensenii*	5306		Sorex araneus	Russia	15	35/8	42/9
	5862		Field mouse	Russia	15	34/8	7/9
	5932		Field mouse	Russia	15	36/8	43/40
	6032		Sorex araneus	Russia	16	33/8	7/9
	5868		Anas acuta	Russia	16	32/8	7/9
	6572		Carrot	Russia	24	31/8	41/41
	H17-36/83	O:12,25		Germany	25	37/8	44/9
	ATCC 33638		Human urin		25	42/21	59/48
*Y. aleksiciae* B	Y332				2	15/1	45/39
	6266			Russia	8	30/18	40/8
	991			Russia	8	29/1	39/8
*Y. mollaretii*	Nr850/89	6,30,47:x:	Water	Germany	9	41/2	51/42
	Nr846/89	62:x:	Water	Germany	9	39/2	48/10
	H279-36/86	O:59		Germany	9	11/2	50/11
	87-36/87				10	40/2	49/10
	H87/82	O:3			10	38/2	46/10
	ATCC 43969		Soil	USA	26	11/2	47/11
*Y. pestis*	91001				11	12/9	8/12
	CO92		Human	USA	11	12/9	52/43
	Pestoides F				11	12/9	8/12
*Y. pseudotuberculosis*	IP 32953	1	Human	France	11	44/9	55/44
	IP 31758	1B	Human	Russia	11	43/9	54/45
	YpIII			USA	11	43/9	53/46
*Y. rohdei*	H274-36/78	O:76		Germany	28	45/19	57/47
	ATCC 43380		Dog feces		29	47/19	60/47
*Y. ruckeri*	Nr 34/85		Fish	Germany	12	13/10	9/13
	H528-36/85				12	13/10	9/13
	H529-36/85			Germany	12	13/10	9/13
	H527-36/85				27	13/10	56/13
	ATCC 29473		Fish		30	13/10	61/49
*Y. similis*	Y239			Germany	31	48/21	62/50
**Total allele number**					**31**	**48/21**	**62/50**

A–NT-nucleotide sequence type, AT-amino acid sequence type.

B–Species identity corrected by 16S-*gyrB *genotype.

In this study, the total number of *Yersinia* strains was 65, covering all *Yersinia* species, and originating from different sources and geographic locations (Table 1). 16S rDNA and *gyrB* sequences from all the above strains were PCR amplified and sequenced. Eight additional sequences of each gene were obtained from publicly available *Yersinia* genomes (http://www.ncbi.nlm.nih.gov). In total 73 16S rDNA and *gyrB* sequences were analysed. The sequences were aligned and adjusted to 750 bp for 16S rDNA and to 838 bp for *gyrB*. Each unique sequence, differing in one or more nucleotide or amino acids sites, was assigned as a distinctive allele, resulting in 31 alleles for 16S rDNA and 48 alleles for *gyrB* or 21 alleles for GyrB (Table 1). The number of the detected alleles was ranged from 1 of 16S rDNA per species (*Y. pestis*, *Y. pseudotuberculosis, Y. enterocolitica* subsp. *palearctica* and *Y. aldovae*) or GyrB (*Y. mollaretii, Y. pestis*, *Y. pseudotuberculosis, Y. similis Y. ruckeri, Y. rohdei* and *Y. bercovieri*) and to 13 alleles of *gyrB* (*Y. intermedia*). The number of allele variants slightly varied from those published previously [29] possibly because of inclusion of more distant strains and/or increasing the lengths of the analyzed fragments.

**Figure 1:**
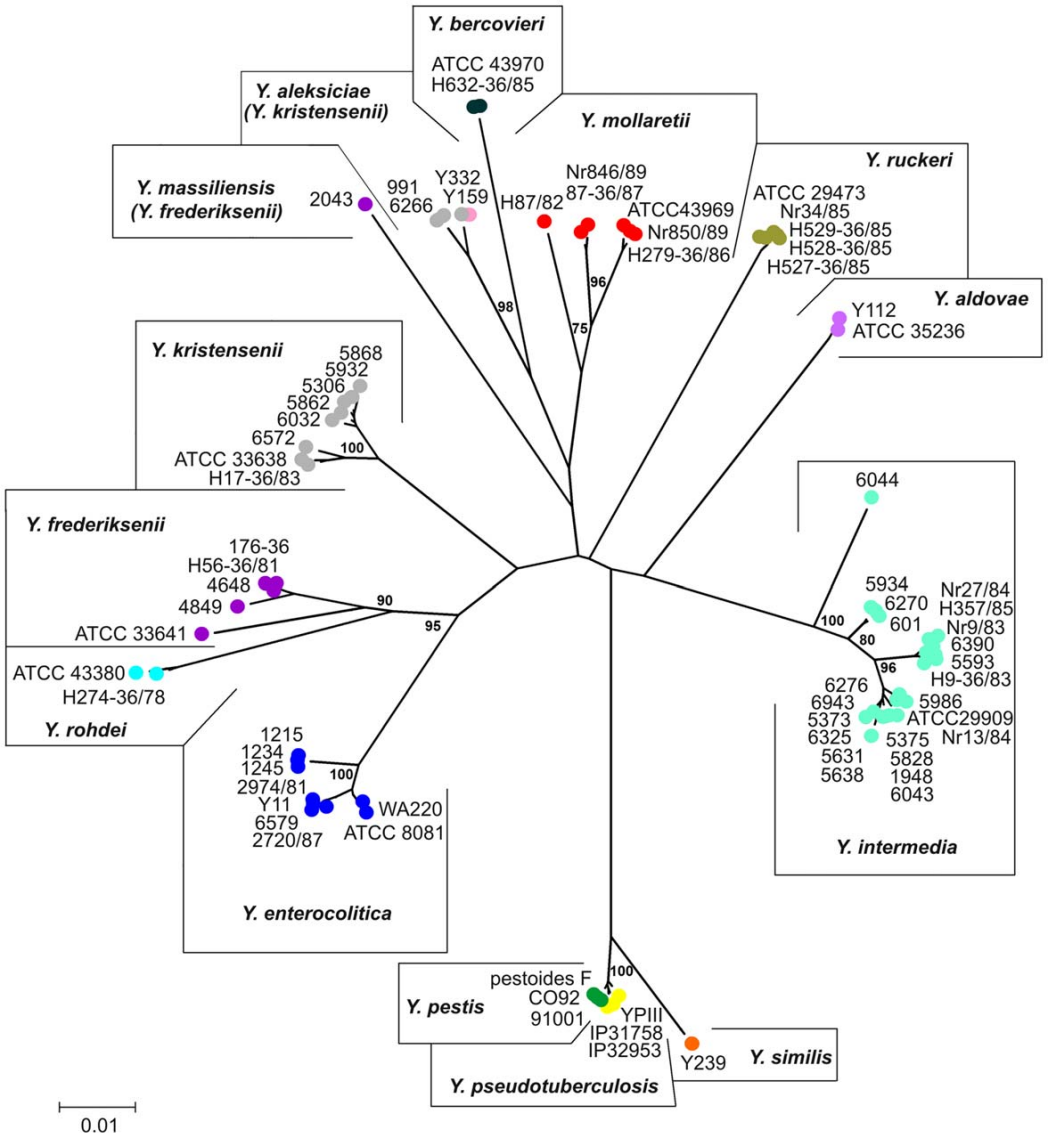
Phylogenetic relationships among 16S rDNA-*gyrB* sequences of *Yersinia*. The unrooted dendrogram was generated using neighbour-joining algorithm. The evolutionary distances were computed using the Kimura 2-parameter method and are expressed in number of base substitutions per site. The percentages of replicate trees in which the associated taxa clustered together in the bootstrap test are shown in nodes.

In order to correctly identify each strain examined, a neighbour-joining tree was constructed from the 16S rDNA-*gyrB* concatenated sequences (Fig. 1). Ten *Yersinia* species (*Y. aldovae*, *Y. bercovieri*, *Y. enterocolitica*, *Y. intermedia*, *Y. mollaretii*, *Y. pestis*, *Y. pseudotuberculosis*, *Y. similis, Y. rohdei* and *Y. ruckeri*) were clearly grouped into relatively distinct clusters. The intraspecies genetic distance means of these species were up to 0.012. *Y. pestis* strains clustered tightly with the *Y. pseudotuberculosis* strains and the distance mean for this group was 0,001. Since, only one *Y. similis* strain was examined, the genetic distance of that species could not be estimated. *Y. similis* is a novel species in *Yersinia*, recently separated from its nearest phylogenetic neighbor *Y. pseudotuberculosis* [3]. As expected, *Y. similis* Y239 was clustered with *Y. pseudotuberculosis* and *Y. pestis*, forming a distinctive long branch. Strains of *Y. enterocolitica* were divided into three groups mainly caused by *gyrB* sequences, while 16S rDNA sequences separated strains into two subspecies (*Y. enterocolitica* subsp. *enterocolitica* and *Y. enterocolitica* subsp. *palearctica*), previously described by Neubauer *et al.*, 2000 [28]. Strain *Y. frederiksenii* 2043 did not group with other five isolates of this species. It branched with *Y. aleksiciae, Y. bercovieri* and *Y. mollaretii.* Based on these results phylogenetic relations and BLAST (data not shown), *Y. frederiksenii* 2043 was more closely related to *Y. massiliensis*. Similar partition was observed for *Y. kristensenii*, three of which (991, Y332 and 6266) diverged from the other eight strains (6572, 8914, H17-36/83, 5868, 6032, 5862, 5306 and 5932) with a genetic distance about 0.055 and clustered with *Y. aleksiciae* Y159, sharing the genetic distance by 0,005. The data definitely indicated that these uncommon strains of *Y. kristensenii* and *Y. frederiksenii* might be members of *Y. aleksiciae* sp. nov. and *Y. massiliensis* sp. nov., since, *Y. aleksiciae* was recently separated from *Y. kristensenii* [1] and *Y. massiliensis* is more closely related to *Y. frederiksenii* [2]. Therefore, these strains were designated as *Y. aleksiciae*-like and *Y. massiliensis*-like, respectively. Based on the 16S rDNA-*gyrB* tree, most *Y. intermedia* clustered together into one of two branches; four Russian strains (6044, 5934, 6270 and 601) were located on the line leading to the rest *Y. intermedia*, shared the intraspecies distances up to 0.007.

Taken together, species identification of *Yersinia* strains based on the 16S rDNA-*gyrB* concatenated tree was in relative agreement with the MLST tree reported previously [29]. Three *Y. kristensenii* strains (991, Y332 and 6266) were designated as *Y. aleksiciae*-like and one *Y. frederiksenii* strain (2043) was as *Y. massiliensis* –like. Six *Yersinia* species (*Y. pestis*, *Y. pseudotuberculosis, Y. bercovieri, Y. ruckeri, Y. rohdei* and *Y. aldovae*) were genetically more homogeneous then the rest of species (*Y. enterocolitica, Y. frederiksenii, Y. mollaretii, Y. intermedia* and *Y. kristensenii*).

### Phylogenetic and recombination analyses of the ompF gene

**Figure 2:**
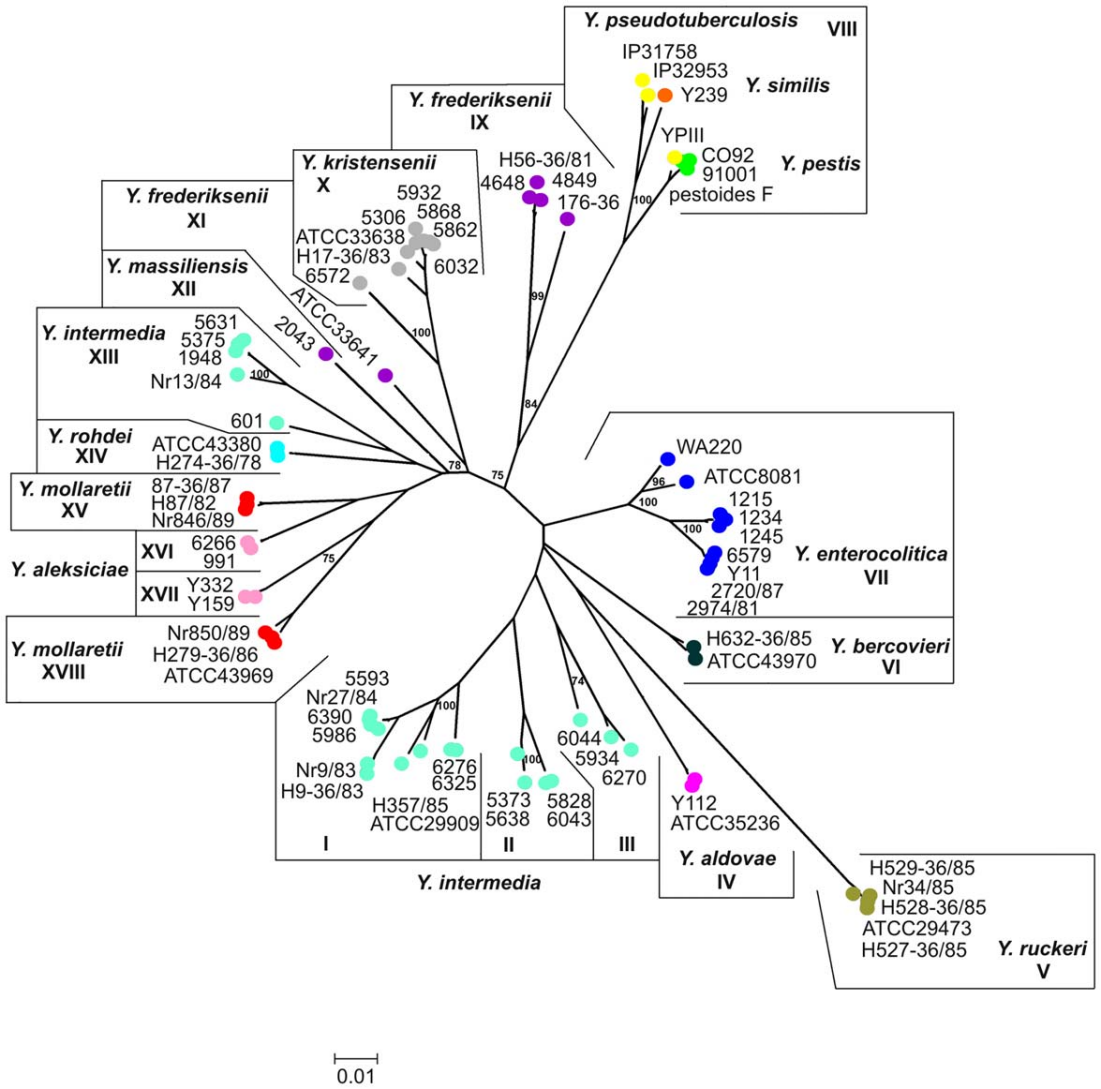
Phylogenetic relationships among *ompF* sequences of *Yersinia*. The unrooted dendrogram was generated using neighbour-joining algorithm. The evolutionary distances were computed using the Kimura 2-parameter method and are expressed in number of base substitutions per site. The percentages of replicate trees in which the associated taxa clustered together in the bootstrap test are shown in nodes.

We investigated phylogenetic relationships and recombination of the *ompF* gene from all *Yersinia* strains (Table 1). The *ompF* gene was amplified, using primers, derived from a CLUSTALX alignment of the published *ompF* nucleotide sequences. 73 complete coding nucleotide sequences of the *ompF* gene were aligned to infer *ompF* phylogenetic tree. We found 62 unique nucleotide alleles of the *ompF* gene (table 1), which clustered into 18 groups on the tree (Fig. 2). Though different algorithms and clustering methods produced similar topologies of the *ompF* tree, phylogenetic clustering of the strains performed by neighbor-joining method with Kimura 2-parameter algorithm gave the highest bootstrap values. With the exception of five species, *Y. similis*, *Y. intermedia*, *Y. mollaretii*, *Y. frederiksenii* and *Y. aleksiciae*, all strains that belong to the same *Yersinia* species were clustered in one group. Five strains of *Y. ruckeri* clustered together in a distinct group V and showed the intragroup distance mean by 0.002 and the largest intergroup genetic distance means from 0.166 to 0.197. It reaffirmed that *Y. ruckeri* has been fairly clonal and genetically the most distant species within the genus [26], [29]. For *Y. enterocolitica* (group VII) the intraspecies genetic distance mean was 0.029, and the groups means of 0.097–0.166. Phylogenetic grouping of *Y. enterocolitica ompF* genes exactly replicated that of 16S rDNA-*gyrB* sequences with division in two subspecies, *Y. enterocolitica* subsp. *palearctica* (Y11, 1234, 2974/81, 6579, 1245, 2720/87, and 1215) and *Y. enterocolitica* subsp. *enterocolitica* (WA220 and ATCC 8081), supported by a high bootstrap value (100%). Interestingly, in both phylogenetic trees, *Y. enterocolitica* subsp. *palearctica* clearly splits into two lines (bootstrap value 100%), one of them was only formed by *Y. enterocolitica* strains (1215, 1234, and 1245) isolated in Russian Far-East. Strains of *Y. kristensenii* formed group X with intragroup distance mean 0.020, and intergroup distance means 0.072–0.183. The *Y. bercovieri* (group VI), *Y. rohdei* (group XIV) and *Y. aldovae* (group IV) were represented by only two strains and the within and between group distance means were up to 0.009 and 0.068–0.188, respectively. The strains of *Y. pestis*, *Y. pseudotuberculosis* and *Y. similis* grouped together (group VIII) with intragroup distance mean of 0.037, and between group distance means being 0.138–0.196. The VIII group splits into two subgroups with bootstrap value of 100%. One of these subgroups included two *Y. pseudotuberculosis* strains IP32953, IP31758 and *Y. similis* Y239, while the other-all *Y. pestis* strains and *Y. pseudotuberculosis* YPIII. This *ompF* tree topology did not correlate with the 16S rDNA-*gyrB* tree branching, possibly indicating interspecies recombination between *Y. pseudotuberculosis* and *Y. similis*, or/and diversification of the *ompF* gene of *Y. pseudotuberculosis* before emergence of *Y. pestis* by adaptive evolution.

The *ompF* sequences of the remaining species, *Y. intermedia*, *Y. mollaretii*, *Y. frederiksenii* and *Y. aleksiciae*, exhibited different phylogenetic relationships and produced incongruent molecular phylogenies with the 16S rDNA-*gyrB* tree. The *Y. frederiksenii* strains, that were genetically distinct and not closely related to each other according to the 16S rDNA-*gyrB* tree, split into three groups; two groups (XI and XII) were presented by single strains, and IX group was by strains with intragroup of 0.043 and intergroup from 0.112 to 0.178 distance means. From previously characterized *Y. frederiksenii* genomic groups [31], *Y. frederiksenii* IX and XI groups of *ompF* could corresponded to genomic groups 1b and 1a, respectively, and XII group (*Y. massiliensis*)-to genomospecies 2. A mixed branching pattern was found in *Y. mollaretii* and *Y. aleksiciae* strains. Two *Y. aleksiciae* strains (Y159 and Y332) grouped together with three *Y. mollaretii* strains (H279-36/85, Nr850/89 and ATCC43969), whereas two others *Y. aleksiciae* strains (991 and 6266)-with three other *Y. mollaretii* (87-36/87, H87/82 and Nr846/89). Therefore, *Y. aleksiciae* (groups XVI and XVII) and *Y. mollaretii* (groups XV and XVIII) strains split into two relatively distinct groups with intragroup distance means up to 0.007, and intergroup distance means of 0.048–0.197. Interestingly, *Y. aleksiciae* recently isolated from *Y kristensenii* was more closely related to *Y. bercovieri* and *Y. mollaretii* than to *Y kristensenii,* and that was confirmed by the *16S rDNA-gyrB* tree. Previously, based on the concatenated *tufA-tufB* tree, *Y. aleksiciae* type strain LMG 22254 was found to be distinct from the *Y. kristensenii* cluster and clearly grouped with *Y. bercovieri* and *Y. mollaretii* [32]. The most genetically heterogeneous was *Y. intermedia* that formed four different groups (I-III, XIII) with intragroup distance means up to 0.039, and with between groups means being 0.076–0.195. Moreover, XIII group, formed by five *Y. intermedia* strains (601, Nr12/84, 1948, 5375 and 5631) was separated from the rest *Y. intermedia* groups by a number of genetic clades.

**Figure 3:**
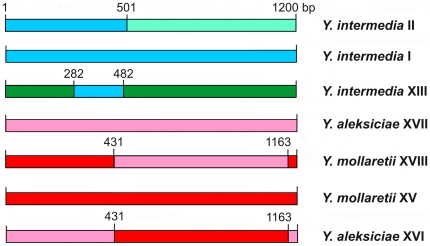
Schematic representation of recombination events with brake-points location in the *ompF* gene of *Yersinia*.

As mentioned above, some species produced incongruent 16S rDNA-*gyrB* and *ompF* phylogenies. A mix branching pattern can be a sign of recombination, whereas in the case of mutation the gene trees look the same [33]. To verify this assumption, we used four tests (RDP, MaxChi, Chimera, and Geneconv) in the RDP3.34 package for investigation of the *ompF* gene of all *Yersinia* groups. We detected four recombination events with brake-points involving three species, *Y. intermedia* (groups I, II, XIII), *Y. aleksiciae* (groups XVI and XVII) and *Y. mollaretii* (groups XVIII and XV) (Fig. 3). From the *ompF* tree, one can suppose that a recombination event between *ompF*s of *Y. aleksiciae* and *Y. mollaretii* occurred twice. In the first case *ompF* of *Y. mollaretii* group XV served as a donor and *ompF* of *Y. aleksiciae* group XVII was a recipient, producing a recombinant *ompF* allele of *Y. aleksiciae* group XVI. And vice versa, *ompF* of *Y. aleksiciae* group XVII served as a donor and *ompF* of *Y. mollaretii* group XV was a recipient, giving a recombinant *ompF* allele of *Y. mollaretii* group XVIII. This explanation comes from comparison of the branch length and sequence diversity of the group members. To our data, interspecies intragenic recombination was detected for the first time in the genus *Yersinia*. We observed a complex pattern of recombination in *Y. intermedia ompF* (groups I, II, XIII). Group I mainly played a parental role in different recombination events, giving *ompF* variants of *Y. intermedia* groups II and XIII; other players of the events were not identified in this analysis. It should be noted, that group I strains are most numerous and widely geographically distributed. So it can be supposed, that this *ompF* variant is more spread and successful in coexistence with mammals including evolutionary newcomers, humans. Acquisition of regions of a successful allele by recombination can be preferred for minor variants (groups II and XIII) when bacterium get into a new niche such as mammals. Interestingly, an extraordinary position of the XIII group on the phylogenetic tree indicates a new origin of the *ompF* gene not represented by any known *Yersinia* species. The fact, that this group includes a human isolate (Nr13/84) may be an evidence of occurrence in new niche, human. Noteworthy, it was extremely difficult to reconstruct a scenario of recombination events for all *Y. intermedia ompF.* This might be a subject of further research, as well as investigation of associations within a specific niche.

It is very interesting to note that one of the brake-points of all recombinant *ompF* is located in the same region (431–501 bp), corresponding to 6-th β-strand of OmpF. The reconstruction of the *ompF* tree for the region 1–501 bp produced very similar branch pattern with that of the 16S rDNA-*gyrB* tree (data not shown). We suspect that significant nucleotide similarity in this region (with the exception of the external loops) reflects a strong selective pressure (purifying selection) due to an important functional role of this region as a zone of 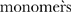 contacts in a porin trimer. This might be an evident example of protein structural constraints.

Examples of the homologous recombination in porin genes have been recognized for some bacteria, mainly for naturally transformable species as *Pseudomonas* (OprD), *Neisseria* (PorB, OmpA), *Chlamydia* (OmpA), and *Leptospira* (OmpL1) [16], [34]–[38]. For these genes different mosaic patterns have been identified. The intragenic recombination has been frequently observed within species due to the transfer of a portion or an entire gene. As a rule, the exchanges occur only in the loop regions and do not affect the transmembrane domains. Moreover, rare cases of interspecies recombination of porin genes have been described in literature. It was suggested that *porB2*, an allele of *porB*, arose in meningococci by interspecies recombination between ancestral pathogenic and commensal *Neisseria* species [39]. Also, an interspecies recombination in *ompA* between a mouse strain of *C. trachomatis* and a horse strain of *C. pneumoniae* was documented [35]. Multiple interspecies recombination patterns were observed among *ompL1* genes, belonging to four different *Leptospira* species [38].

The phylogenetic analysis of *ompF* sequences placed most of the *Yersinia* strains in the same line assigned by *16S rDNA-gyrB* tree with the exception of six species, *Y. pseudotuberculosis*, *Y. similis*, *Y. frederiksenii*, *Y. intermedia*, *Y. mollaretii*, and *Y. aleksiciae.* The incongruence of *ompF* and *16S rDNA-gyrB* trees indicated the inter- and intraspecies recombination. Despite extensive recombination events in the *Yersinia ompF* genes, this seems to happen not so often to remove all phylogenetic signals.

### Adaptive evolution of the ompF gene in Yersinia

**Figure 4:**
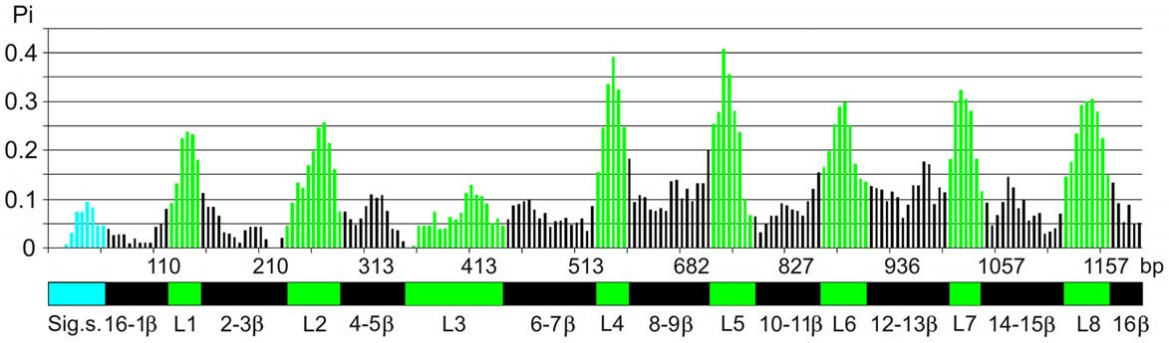
Nucleotide divergence (Pi) in 73 *ompF* sequences. The regions predicted to correspond to the external loops (L1–L8) are colored green, regions putatively exposed to the periplasm and predicted transmembrane strands (1-16β) are indicated by black shading, the signal sequence (Sig.s.) is colored blue.

As it was shown above, the *ompF* gene of *Yersinia* is more divergent than the 16S rDNA and *gyrB* genes. The nucleotide diversity for all *ompF* genes (0,131±0,005) is twofold higher than for housekeeping genes (0,051±0,004). The common alignment of 73 *ompF* sequences contain 40% (479/1200 bp) of polymorphic nucleotide sites, which distributed strikingly nonrandom and formed hypervariable and conserved regions (Fig. 4). We have divided yersinia's *ompF* gene into 18 regions, according to domain organization of *Escherichia coli* OmpF protein [9]. Loops L2, L4–L7 were characterized by nucleotide deletions and/or insertions. Comparative analysis of surface-exposed loops exhibited significant heterogeneity of L4 and L5 (46±4.5%). The highest homology was conserved in L3 (8.2±1.6%). The same nonrandom heterogeneity with characteristic conserved regions forming the β-barrel structure of the proteins, and variable regions, making up the putative surface-exposed loops, has been shown in some other porins [39], [40].

To estimate deviation in codon usage, the codon adaptation index (CAI) was calculated for the *ompF* gene. CAI is a measure of the relative adaptiveness of the codon usage of a gene towards the codon usage of highly expressed genes of that organism: the higher the index value, the greater the codon usage bias [41]. As a reference for highly expressed genes, we used the 27 concatenated ribosomal genes for ten *Yersinia* species. The genes of the ribosomal proteins had a CAI value from 0.52 to 0.56 for all species, but CAI values for the *ompF* gene were higher (from 0.64 to 0.75). Therefore, there is a strong codon usage bias in the *ompF* gene in all *Yersinia* species, as expected for highly expressed genes. This is another reason to assume that the high level of *ompF* transcription may be also responsible for nonrandom heterogeneity in the gene.

**Figure 5:**
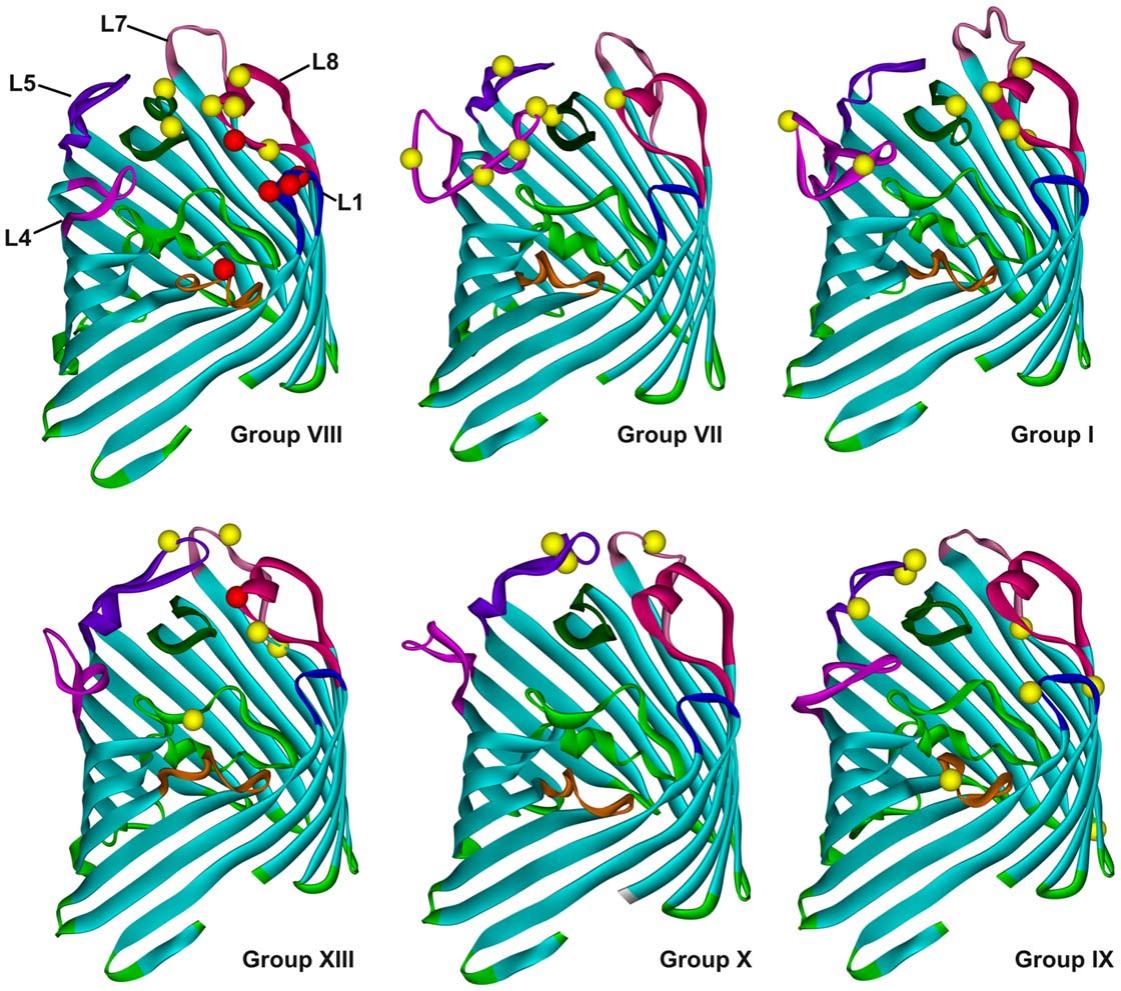
Location of positively selected sites in OmpF porins of *Yersinia*. Group VII-*Y. enterocolitica* WA220; Group XIII-*Y. intermedia* 1948; Group IX-*Y. frederiksenii* 4648; Group I-*Y. intermedia* ATCC 29909; Group X-*Y. kristensenii* 5868; Group VIII-*Y. pseudotuberculosis* IP 31758. Sites that show positive selection (P<0.05) are depicted as yellow spheres and (P<0.01)-as red spheres.

To determine how the level of selective constraint varies along the *ompF* gene, we estimated the numbers of synonymous substitutions per synonymous site (dS) and nonsynonymous substitutions per nonsynonymous site (dN) and calculated the dS/dN ratio for the *ompF* gene. If purifying selection has occurred, a gene has a dS/dN>1. Absence of selection should generate dS/dN = 1. A ratio dS/dN<1 indicates diversifying selection or accelerated evolution [42], [43]. We excluded *Yersinia* groups with recombination events from analysis and dealt only with six *ompF* groups of *Yersinia* (VII, VIII, IX, I, X, XIII). The dS/dN ratio was calculated as an average over all of the codon sites in each *ompF* group using the Nei-Gojobori method by MEGA 4 of Jukes-Cantor model. Statistical significance was tested by Codon-based Z-test. For all groups we detected approximately identical dS/dN means from 4.224 to 5.748 with p<0.05 of purifying selection. Thus, *ompF* gene is under strong purifying selection in all six *Yersinia* groups. Neilsen and Yang method [44], compiled in Sitewise likehood ratio estimation programme [45], was used to identify the sites with the evidence of positive selection in selected *ompF* groups. The porin protein structures for these groups have been simulated and sites with weak or strong positive selection have been located on the models (Fig. 5).

When these selected sites were mapped onto three-dimensional structural models, it becomes clear that the majority fell within regions predicted to encode surface-exposed loop regions. It is important to note that these sites were located in different surface loops of analyzed *Yersinia* groups. For example, three residues in putative loop L1 were shown to be under strong selection in the group VIII, whereas there is no evidence of positive selected sites in putative loop L1 for groups VII, XIII, I and X. Smith N.H. observed unlike distribution of positive selected regions in *porB* genes in *N. meningitides* and *N. gonorrhoeae* [46]. Authors explained this by differences in the immune response to these two organisms. The impact of diversifying selection on *ompC*, *ompF*, *lamB* and *fhuA* omp's genes of *Escherichia* and *Shigella* [47], [48], *ompC, ompS1* and *ompS2* genes of *Salmonella* [49] has been demonstrated. Authors proposed that positive selection in *omp* genes may be an important mechanism that facilitates adaptation of bacterial pathogens allowing them to escape recognition by the host immune system, phages and penetration of antibiotics.

Our analyses demonstrated that the *Yersinia ompF* gene has evolved with nonrandom mutational rate under purifying selection in overall. However, the surface loops of the OmpF porin contain sites subjected to positive selection. Interestingly, such sites are located in different surface loops in different *Yersinia* species. We suppose that the *ompF* genes of different *Yersinia* species have evolved under individual constraints associated with unlike environmental challenges. Existence of both positive selection and recombination in porin genes has previously been reported for Neisseria *porB* and *porA* genes [37], [50] as well as for *ompA* from *Chlamidia* [35] and *Wolbachia* [51]. In case of *Yersinia ompF* gene we consider that horizontally acquired fragments of some surface loops may be fixed by positive selection in process of species adaptation to new ecological niches. Such recombinant genes might supply their new hosts with benefits allowing to escape a deadly response of the immune system as well as lethal attacks of phages and antimicrobials. This might be more easily achieved by gene recombination rather than by random mutations. Moreover, these mechanisms seem to operate in evolution of porins genes of all taxonomic groups.

#### Conclusion

Genetic diversity of outer membrane proteins might result from bacterial adaptation to different ecological niches. Porins are surface exposed and their structure strongly reflects the history of multiple interactions with the environmental changes in their ecological niches. The evolution of the *ompF* gene of *Yersinia* clearly demonstrates a combination of diversifying selection (recombination and positive selection) and function-structure constraint (translational selection and purifying selection). The data can be important for clarification the role of porin's surface exposed loops on bacterial adaptation and development of broad-spectrum *Yersinia* vaccine antigens and serological methods of diagnostics.

## Materials and Methods

### Bacterial strains, growth conditions, and DNA isolation

A total of 65 *Yersinia* strains from the collections of Max von Pettenkofer Institute (Munchen, Germany) and Research institute of epidemiology and microbiology, Siberian branch of Russian academy of medical sciences, (Vladivostok, Russia) were used in this study. Strain selection was intended to include strains of all known *Yersinia* species with a high degree of diversity. All strains were grown overnight at 30°C or 37°C under aerobic conditions on LB medium. Bacterial DNA was isolated from overnight cultures of the selected strains using Genomic DNA Purification Kit (Fermentas, EU). The DNA concentration was determined by agarose gel electrophoresis. The gels were scanned and the signals were analyzed with the VersaDoc 4000 MP system (Bio-Rad Laboratories AG, Switzerland). Additionally, eleven *Yersinia* strains for which the genome sequences are available on the GeneBank of NCBI website were analyzed.

### PCR amplification and DNA sequencing

PCR amplification of 16S rDNA gene from all strains of *Yersinia* was performed using the primers, BF-20 (5′–ATCACGCGTAAAAATCT-3′) and BR2-22 (5′-CCGCAATATCATTGGTGGT-3′). The expected amplicon size was 1500 bp. The part of *gyrB* gene was amplified using primers YgyrF (5′-CCCACTTTATACCT-3′) and YgyrR (5′-CCCACTTTATACCT-3′). The expected amplicon size was 980 bp. The *ompF* gene was amplified using primers Fcds-F (5′-CCCACTTTATACCT-3′) and Fcds-R (5′-CCCACTTTATACCT-3′). These were designed by aligning sequences of *ompF* genes of *Y. enterocolitica* 8081 (AM286415), *Y. intermedia* ATCC 29909 (AALF02000006), *Y. mollaretii* ATCC 33641 (NZ_AALD02000003) and *Y. frederiksenii* ATCC 33641 (NZ_AALE02000015). The expected amplicon size was 1100 bp. PCR conditions for all genes were as follows: initial denaturation at 95°C for 5 min followed by 30 cycles each at 94°C for 30 s, 55°C for 30 s, 72°C for s and a final extension step at 72°C for 5 min. PCR products were evaluated on a 1,5% agarose gel stained with ethidium bromide. Unincorporated primers and dNTPs were removed from PCR products with NucleoSpin® Extract II kit (Macherey-Nagel). Purified DNA was sequenced using the dideoxynucleotide chain-termination method with fluorescent ddNTPs from Applied Biosystems on an ABI 310 Prism automated DNA sequencer, in accordance with the manufacturer's instructions. Sequence data for the appropriate loci from *Y. bercovieri* ATCC 43970 (NZ_AALC00000000), *Y. enterocolitica* 8081 (NC_008800), *Y. frederiksenii* ATCC 33641 (NZ_AALE00000000), *Y. intermedia* ATCC 29909 (NZ_AALF00000000), *Y. mollaretii* ATCC 43969 (NZ_AALD00000000), *Y. pestis* 91001 (NC_005810), CO92 (NC_003143), Pestoides F (NC_009381), *Y. pseudotuberculosis* IP 31758 (NC_009708), IP 32953 (NC_006155), YPIII (NC_010465), *Y. rohdei* ATCC 43380 (NZ_ACCD00000000), *Y. kristensenii* ATCC 33638 (NZ_ACCA00000000), *Y. ruckeri* ATCC 29473 (NZ_ACCC00000000), *Y. aldovae* ATCC 35236 (NZ_ACCB00000000) were obtained from GenBank (http://ncbi.nlm.nih.gov) and analyzed together with other *Yersinia* isolates (Table 1).

### Comparative sequence analysis and phylogeny inference

Nucleotide sequence data from forward- and reverse-strand chromatograms were assembled into single contiguous sequences using the Vector NTI Advance 9.1.0 software. Sequences were aligned by ClustalW 2.0.10 [52]. MEGA version 4.1 [53] was used to calculate genetic distances between sequences and to produce phylogenetic trees. To construct the tree from nucleotide sequences, all three coding positions were examined and the Neighbour-Joining model with Kimura 2-parameter method [54] was applied. The reliability of the inferred trees was assessed using the bootstrap test (1000 replications) [55]. Alignment gaps were excluded using function “Pairwise Deletion” from all analyses.

### Evolution analyses

Nucleotide divergence (Pi) along *ompF* sequences was determined by DnaSP v5 [56] using Sliding window with length of 20 and step size of 7. Adaptive evolution of *ompF* gene was calculated as proportion of synonymous (silent; ds) and non-synonymous (amino acid-changing; dn) substitution rates in MEGA 4 using the Nei-Gojobori method with Jukes-Cantor correction and SLR [45] software. Recombinant *ompF* sequences were detected with the RDP v3.34 software [57] using four automated recombination detection methods including RDP [58], Genconv [59], Chimaera [60], Maximum Chi Square [60], [61]. For the RDP method, internal reference sequences were used, the window size was set to 20, and 0–100 sequence identity was used. For both the MaxChi and the Chimera methods, the number of variable sites was set to 40. For the GENCONV method, we used standard settings. A maximum P value of 0.01 and a Bonferroni correction were used. Results were then checked by visual inspection. CAI index was calculated by CodonW 1.3 (ftp://molbiol.ox.ac.uk/cu/codonW.tar.Z) software for 11 *Yersinia* species (*Y. pestis* CO92, *Y. pseudotuberculosis* IP32953, *Y. enterocolitica* ATCC 8081, *Y. intermedia* ATCC 29909, Y. rohdei ATCC 43380, *Y. kristensenii* ATCC 33638, *Y. frederiksenii* ATCC 33641, *Y. mollaretii* ATCC 43969, *Y. ruckeri* ATCC 29473, *Y. bercovieri* ATCC 43970, *Y. aldovae* ATCC 35236). As a reference for highly expressed genes, we used the 26 concatenated ribosomal genes for each organism.

### Nucleotide sequence accession numbers

The novel sequences determined in this study have been deposited in GenBank under accession no. GQ421361-GQ421424; FJ641877-FJ641894; 146 HM142614-HM142721.
